# Precision of Ultrasound-Guided versus Anatomical Palpation-Guided Needle Placement of the Ulnar Nerve at the Cubital Tunnel: A Cadaveric Study

**DOI:** 10.3390/healthcare11111603

**Published:** 2023-05-30

**Authors:** Carlos López-de-Celis, César Fernández-de-Las-Peñas, Miguel Malo-Urriés, Isabel Albarova-Corral, José L. Arias-Buría, Albert Pérez-Bellmunt, Jacobo Rodríguez-Sanz, Vanessa González-Rueda, Sergio Borella-Andrés

**Affiliations:** 1 Faculty of Medicine and Health Sciences, Universitat International de Catalunya, 08028 Barcelona, Spain; 2 ACTIUM Functional Anatomy Group, 08028 Barcelona, Spain; 3 Fundació Institut, Universitari per a La Recerca a l’Atenció, Primària de Salut Jordi Gol i Gurina (IDIAPJGol), 08028 Barcelona, Spain; 4 Department of Physical Therapy, Occupational Therapy, Rehabilitation and Physical Medicine, Universidad Rey Juan Carlos, 28922 Alcorcón, Spain; 5 Health Sciences Faculty, Department of Physiatry and Nursery, University of Zaragoza, 50009 Zaragoza, Spain

**Keywords:** needle, ultrasound, ulnar nerve, cubital tunnel, accuracy, palpation

## Abstract

Percutaneous electrical stimulation has been performed for years with only the assistance of anatomical landmarks. The development of real-time ultrasonography guidance has improved the precision and safety of these percutaneous interventions. Despite ultrasound-guided and palpation-guided procedures being performed routinely for targeting nerve tissues in the upper extremity, the precision and safety of these techniques are unknown. The aim of this cadaveric study was to determine and compare the precision and safety of ultrasound-guided versus palpation-guided needling procedure with and without the handpiece of the ulnar nerve on a cadaveric model. Five physical therapists performed a series of 20 needle insertion tasks each (*n* = 100), 10 palpation-guided (*n* = 50) and 10 ultrasound-guided (*n* = 50) on cryopreserved specimens. The purpose of the procedure was to place the needle in proximity to the ulnar nerve at the cubital tunnel. The distance to target, time performance, accurate rate, number of passes, and unintentional puncture of surrounding structures were compared. The ultrasound-guided procedure was associated with higher accuracy (66% vs. 96%), lower distance from needle to the target (0.48 ± 1.37 vs. 2.01 ± 2.41 mm), and a lower frequency of perineurium puncture (0% vs. 20%) when compared with the palpation-guided procedure. However, the ultrasound-guided procedure required more time (38.33 ± 23.19 vs. 24.57 ± 17.84 s) than the palpation-guided procedure (all, *p* < 0.001). Our results support the assumption that ultrasound guidance improves the accuracy of needling procedures on the ulnar nerve at the cubital tunnel when compared with palpation guidance.

## 1. Introduction

Cubital tunnel syndrome is a pain condition associated with compression of the ulnar nerve around the cubital tunnel [1]. Cubital tunnel syndrome is the second most prevalent compressive neuropathy of the upper extremity, with an incidence of 19 to 25 cases per 100,000 person-years [2], and a prevalence of 2–6% in the general population [3]. In the early stages of cubital tunnel syndrome, symptoms include paraesthesia, dysesthesia, and numbness in the 4–5th fingers and ulnar side of the hand [4]. As the disease progresses, weakness and atrophy of the flexor carpi ulnaris, ulnar flexor digitorum profundi, hypothenar, and intrinsic muscles of the hand occur, affecting professional and daily life activities [5]. The most common sites of entrapment of the ulnar nerve include the arcade of Struthers, the medial septum, the deep flexor-pronator aponeurosis, the Guyon’s canal, and the cubital tunnel [6]. The cubital tunnel is formed by the retro-condylar groove and is covered by the Osborne ligament.

The treatment of cubital tunnel syndrome is divided into surgical and conservative. Surgery, including several types of decompression, transposition, or medial epicondylectomy is recommended for severe and persistent symptoms, with motor impairment, and when non-operative treatments have failed [5]. However, a conservative approach, including avoidance of sustained elbow flexion, nonsteroidal anti-inflammatory drugs, steroid injections, or physical therapy, is recommended as the first-line therapy in the early and mild-to-moderate stages [7]. Physiotherapy is one of the most common and effective conservative treatments for peripheral neuropathies [8]. To date, various techniques of physiotherapy, including percutaneous electrical nerve stimulation, have proven to be potentially effective in the treatment of cubital tunnel syndrome [9,10,11,12]. Percutaneous electrical nerve stimulation is an emerging technique that applies electrical biphasic current for pain treatment. Electrical stimulation has been used for the treatment of neuropathic pain since the 1970s [13], but its use for cubital tunnel syndrome has not been disseminated. Neuromodulation evokes neural activity producing a reversible nerve block with a selective reduction of pain sensitivity while preserving other nerve functions [14]. To optimize the therapeutic effects, highly targeted applications can be achieved by placing neuromodulation on specific nerve fascicles [15]. The success of percutaneous electrical stimulation approaches requires precise needle placement [16]. In fact, inaccurate needle positioning is the main cause of loss of effectiveness and risk of adverse events [17].

Traditionally, several needling interventions have been performed with the only assistance of palpation. However, the development of imaging procedures to guide needling techniques has decreased reliance on tactile sensations. Ultrasound provides real-time visual guidance that is consistently reported to improve accuracy and reduce the adverse effects of invasive techniques compared with unassisted procedures [18]. Thus, ultrasound guidance has become the standard for several invasive techniques. Despite ultrasound-guided and palpation-guided invasive being performed routinely, there is almost no evidence comparing ultrasound-guided procedures with palpation-guided procedures. Therefore, the purpose of this study was to compare ultrasound-guided with palpation-guided procedures for needling placement at the ulnar nerve in the cubital tunnel in terms of accuracy, performance time, number of passes, or incidence of unintentional puncture of surrounding structures.

## 2. Methods

### 2.1. Study Design

The study obtained the Ethics Committee approval (CBAS-2021-09; Comitè d’Ética de Recerca, International University of Catalonia). Five physical therapists who specialized in invasive needling interventions participated. Written informed consent was obtained from participants.

A cadaveric model was used to present a completely realistic situation in terms of anatomy. Frozen specimens were stored under refrigerated conditions (−20 °C) and thawed to ambient temperature prior to the procedure to ensure normal tissue characteristics. The cadaveric model was placed in a similar position to a clinical situation, with optimized ergonomics, including handling of the transducer and needle.

Participants received a 10 min instructional and practical standardized session to understand the study’s purpose and familiarize themselves with the procedure.

### 2.2. Procedure

**Figure 1:**
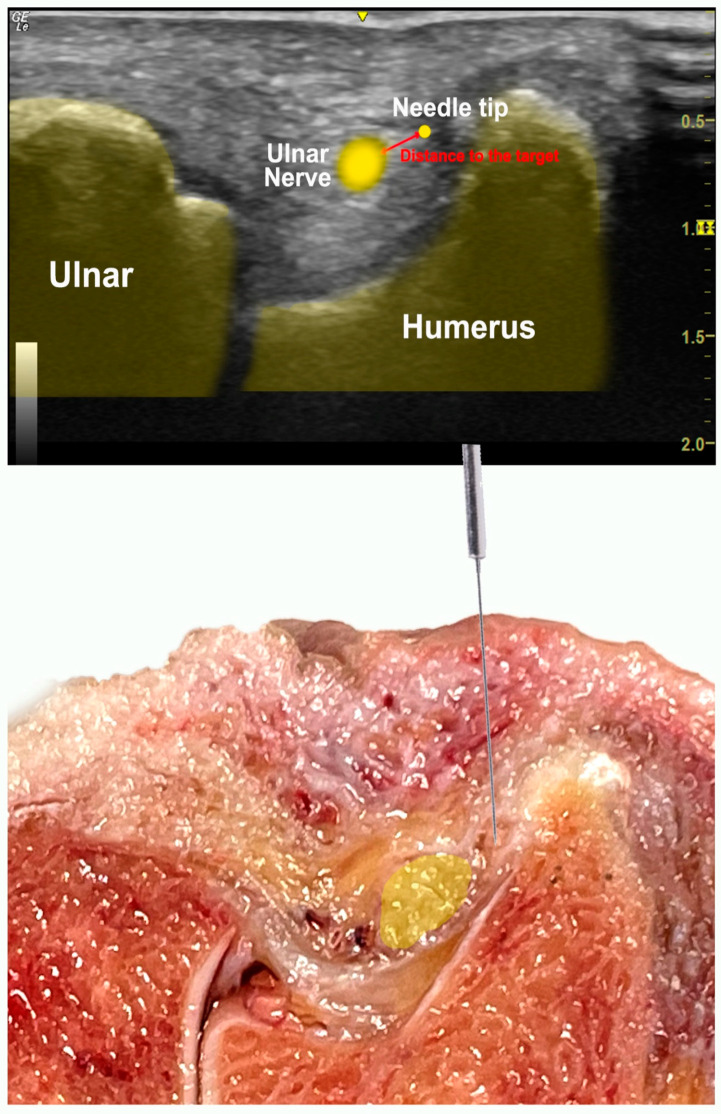
Ultrasonographic and cadaveric scheme of the procedure of the ulnar nerve in the cubital tunnel. The measurement of the distance between the needle tip and the ulnar nerve is shown in red.

Participants were instructed to place the needle in contact with the most superior and medial point of the ulnar nerve in the cubital tunnel of the cadaveric model (Figure 1). The needle tip should be positioned as closed as possible to the epineurium to avoid passing through it (the target proximity task) [19]. As many needles passes as necessary were allowed until the final placement was accurate.

This procedure was conducted under the following two conditions: palpation-guided and ultrasound-guided. Each therapist completed a total of 20 tasks (10 under palpation-guiding and 10 ultrasound-guided) with a short wash-out break after each procedure and 5 min break rest after 10 attempts to prevent fatigue [20,21].

#### 2.2.1. Palpation-Guided Procedure

**Figure 2:**
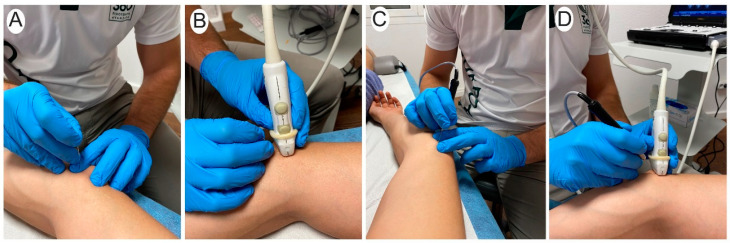
Procedure of the needling interventions guided by palpation (**A**,**C**) and guided by ultrasound (**B**,**D**). The procedure was performed without the handpiece (**A**,**B**) and with the handpiece (**C**,**D**).

Participants were asked to complete the task with the sole guidance of their palpatory skills. Initially, participants should identify the ulnar nerve in the cubital tunnel. The needle was inserted with the dominant hand between the medial epicondyle and olecranon (Figure 2A–C). Each participant first performed the 10 palpation-guided approaches and then the 10 ultrasound-guided approaches to avoid pre-visualization of the ultrasound that could help palpation-guided approaches.

#### 2.2.2. Ultrasound-Guided Procedure

Participants were asked to complete the task under the guidance of ultrasound imaging. A LOGIQ e R8 (General Electric Healthcare) ultrasound equipment with a 4 to 12 MHz linear transducer was used. The ultrasonographic image was pre-calibrated and optimized for parameters (frequency, depth, gain, and focus) by the researchers in a standardized manner to allow participants to focus on the technique. The depth was 2 cm with a frequency of 10 MHz. The focus was centered on the ulnar nerve, and the gain was adapted according to the characteristics of the subject to obtain a sharp image. To identify the ulnar nerve, the probe, held in the non-dominant hand, was positioned in the transversal plane of the cubital tunnel, between the medial epicondyle and the olecranon. Once an optimal visualization of these structures was obtained, the needle was inserted with the dominant hand from the medial side (Figure 2B–D).

Both conditions were performed with a filiform needle (5 palpation-guided and 5 ultrasound-guided; Figure 2A–C) and with a filiform needle inserted into a handpiece of percutaneous electrolysis equipment (5 palpation-guided and 5 ultrasound-guided; Figure 2B–D). The use of the handheld was randomly selected following a computerized random assignment list. All tasks were performed with sterile stainless-steel filiform solid needles (0.30 × 40 mm, Agupunt, Barcelona).

Following each needle placement, two experienced researchers registered: the distance of the tip of the needle to the target (in millimeters) (Figure 1); if the epineurium of the ulnar nerve was punctured; in case of puncture of the ulnar nerve, the total distance was recorded (in millimeters); the total time needed for completing the needling procedure (in seconds); the total number of needle passes (each time a therapist advanced the needle after a change of direction was considered one pass) [19], and the needle length outside the body (millimeters). An attempt was considered accurate if the needle tip was placed less than 3 mm from the target was properly achieved [20].

Feedback was not provided at the end of each attempt to not interfere with subsequent insertions [22]. It has been demonstrated that when practitioners receive feedback after a procedure, they improve their performance [23].

At the end of all insertions (*n* = 20), each therapist was asked to fulfill a standardized questionnaire to quantify the workload required for each procedure [24]. This questionnaire evaluates the perceived amount of work (mental, physical, and temporal demands, perceived performance, effort and level of frustration on a 0–20 points scale), their own ability (0–10 points scale) for each procedure, and the therapist had to select their preferred modality (ultrasound-guided or palpation-guided).

### 2.3. Statistical Analysis

Data were analyzed with IBM SPSS statistics 22.0 software. Descriptive data were expressed as total number, percentage, mean and standard deviation (SD). The normal distribution of the variables was analysed using the Kolmogorov-Smirnov test. Comparative analysis between palpation-guided and ultrasound-guided procedures for quantitative variable was performed using an analysis of variance (ANOVA) test. The chi-square (χ2) test was used to assess the differences in nominal variables. Answers of the questionnaire were analysed using Wilcoxon signed-rank test for non-parametric paired observations. The significance level was set at 0.05.

## 3. Results

Demographic data from the participants and overall data from the procedures are shown in Table 1. The comparison of the measurements between palpation-guided and ultrasound-guided procedures is detailed in Table 2. The ultrasound-guided procedure significantly increased the accuracy rate from 66% with the palpation-guided procedure up to 96% (*p* < 0.001) and also decreased the distance from the tip of the needle to the ulnar nerve (0.48 ± 1.37 vs. 2.01 ± 2.41 mm, *p* < 0.001). Further, the ultrasound-guided procedure was also associated with a lower frequency of perineurium puncture (0%) when compared with the palpation-guided procedure (20%, *p* = 0.001). Nevertheless, the ultrasound-guided procedure required significantly (*p* = 0.001) more time (38.33 ± 23.19 s) than the palpation-guided procedure (24.57 ± 17.84 s). No significant differences between procedures in the number of passes (*p* = 0.463) were found.

Table 3 shows the parameters evaluated during the procedures performed only with the needle or with the needle place into the handpiece. No significant differences were observed between procedures with or without handpiece (all, *p* > 0.127) except for millimeters of the needle outside of the skin (*p* = 0.002).

The results of each category of the self-perceived questionnaire are shown in Table 4. No significant differences between palpation-guided and ultrasound-guided procedures in the self-perceived questionnaire (all, *p* > 0.317) were observed. Nevertheless, all therapists preferred the ultrasound-guided technique without the handpiece as the most comfortable procedure.

## 4. Discussion

Accurate needle placement is the first requirement of multiple interventional procedures performed by different health professionals, including percutaneous electrical stimulation. In fact, inaccurate needle placement is a major cause of loss of effectiveness and risk of adverse events [25]. Traditionally, several invasive techniques have been performed with the guidance of anatomical landmarks or with the assistance of palpation. However, ultrasound guidance has been demonstrated to improve the performance of invasive techniques [26]. Although ultrasound-guided procedures for the ulnar, radial, and median nerve in the elbow are frequently used in clinical practice, there is a lack of evidence regarding their validity and safety [27]. The aim of this study was to compare the performance of ultrasound-guided versus palpation-guided invasive procedures on the ulnar nerve in the cubital tunnel on a cadaveric model. The results of our cadaveric study showed that the ultrasound-guided procedure significantly increased accuracy with a lower unintentional puncture of the ulnar nerve perineurium when compared with the palpation-guided procedure. The accuracy of the ultrasound-guided procedure was 96%, whereas the accuracy of the palpation-guided procedure reached 66%. However, the ultrasound-guided procedure required significantly more time than the palpation-guided.

In this study, an ultrasound-guided procedure significantly decreased the distance from the needle to the targeted tissue supporting an accurate placement of the tip of the needle. Consistent with our results, ultrasound-guided procedures have consistently been demonstrated to improve accuracy for other peripheral nerves [18,26,28]. In fact, previous studies using different systems of ultrasound-guidance with achieved accuracy rates between 1.50–3.27 mm with different phantoms [19,29,30]. These results are slightly lower than those found in our study; however, the task is not the same, so a direct comparison would not be appropriate. Furthermore, performing the task on a cadaveric specimen implies a level of anatomical realism, which could favor the performance of professionals with a high level of experience. On the other hand, performing the task on simulated models such as phantoms could be more favorable for trainees.

Regarding the safety of the technique, different techniques of neuromodulation have demonstrated its clinical effectiveness; however, there is a lack of evidence about its safety [31]. Security of invasive techniques is especially important in patients that are anticoagulated [32]. In the current cadaveric study, the ultrasound guidance achieved a reduction in the frequency of unintentional puncture of the ulnar nerve perineurium (0% compared to 20% palpation guidance). Previous studies of regional anesthesia have also demonstrated greater safety of ultrasound-guided interventions [17]. To achieve better accuracy and safety, the ultrasound-guided procedure required significantly more time compared to palpation guidance. In contrast, some previous studies have found that ultrasound guidance can reduce the time of the procedure [21,28,33]. These differences may be due to the fact that ultrasound guidance allows real visual feedback on the positioning of the needle, so the therapist can correct and refine the technique until the exact point is reached, which is more time-consuming but also more accurate and safer.

Regarding the workload perceived by the participants, at the end of the 20 punctures, each participant was asked to fulfill a questionnaire evaluating the perceived amount of work for each procedure [24]. Previous studies found high frustration levels and lower assessment of their own ability when the palpation-guided techniques were used [24]. In our study, no significant differences between the palpation-guided and ultrasound-guided procedures were observed. Nevertheless, all therapists preferred the ultrasound-guided technique without the handpiece.

The target of the study was to insert the needle close to the ulnar nerve in the cubital tunnel. At the elbow, the ulnar nerve passes at the retrocondylar groove, posterior to the medial epicondyle, in the cubital tunnel. The cubital tunnel is bordered medially by the medial epicondyle, laterally by the olecranon process, deeply by the medial collateral ligament of the elbow, and superficially by Osborne´s band. This is a point of great clinical relevance as the nerve can be subjected to compression, traction, and friction forces during normal elbow movements and static postures. Due to the proximity of the nerve to the bone and the small amount of soft tissue padding at the elbow, the ulnar nerve is vulnerable to external pressure as well as to other dysfunctions [34]. Depending on nerve strain and its duration, inflammation, and swelling can be produced, rendering the ulnar nerve vulnerable to ischemia [35].

The results of the current study should be considered by practitioners performing invasive neuromodulation techniques, especially for patients with cubital tunnel syndrome. It should be noted that surgical treatment is required for severe and persistent symptoms, motor weakness, or failure of non-operative treatments [5]. However, conservative options are recommended in mild-to-moderate cases where there is no motor weakness. Several physical therapy techniques have been used in the management of cubital tunnel syndrome, e.g., manual therapy, neurodynamic interventions, or electrical techniques [9,10,11,12]. Although peripheral neuromodulation has been used for more than 50 years and the technology has been available for decades [13], its evolution over the last two decades has made it a key treatment option for pain management [36]. Clinical success and specificity of neuromodulation depend on the correct placement of the electrodes or needles in close proximity to the neural interface but without damaging the epineurium. Improved imaging techniques to guide the placement of the needle, such as ultrasonography, have rendered the procedure more secure and effective, with higher patient satisfaction, lower incidence of unintentional puncture of the target, and, thus, more widely used [37]. Ultrasound provides a real-time visual aid to guide different techniques in an economical, safe, radiation-free, non-invasive, and portable way. This could be especially important in anatomical areas of major risk or requiring high precision. Because of these advantages, ultrasound guidance has become a mainstay of several invasive techniques.

Our study has some limitations that should be taken into account when interpreting the results. First, it was preferred to always start with the palpation-guided procedure, as it could have existed a learning bias if the ultrasound-guided procedure had been performed first. The visual feedback of ultrasound has been demonstrated to be a crucial factor in the learning process [24]. This fixed order (palpation followed by ultrasound-guided) may induce bias due to fatigue in the latter tasks. To avoid fatigue, rest times were introduced after each attempt and after each block of 10 attempts. Second, procedures were applied by five experienced therapists. Third, while the cadavers were thawed at room temperature, the body temperature did not match the temperature of a living person. Lower temperatures result in increased tissue stiffness, which could potentially affect the accuracy of the manual palpation method when inserting the needle. Future studies could determine intra- and inter-operator reliability and differences between therapists with different levels of training and experience. Ultrasound guidance could be a relevant teaching tool to overcome inexperience and lack of confidence, as well as offering a tri-dimensional knowledge of anatomy [20]. Third, our study was conducted on a human cadaveric sample. Most previous studies have been performed on synthetic phantoms or animal cadaveric samples, enabling multiple punctures under identical conditions [38]. However, due to their composition or structure, they are generally limited in terms of clinical reality [24]. In fact, previous authors consider that further research in human rather than animal tissue models is necessary [19]. Our study covers this need previously proposed. The human cadaveric model is completely realistic in anatomical terms and enables multiple punctures. However, our results must be interpreted considering that it was a cadaveric sample and not an in vivo sample. Finally, future studies should confirm these results considering the proposed limitations, as well as analyze the accuracy of techniques applied in other regions of the ulnar nerve, as well as in other tissues.

## 5. Conclusions

Although the palpation-guided procedure exhibits acceptable levels of precision, the ultrasound-guided procedure showed significant improvements in terms of accuracy and safety. However, to achieve these improvements, the ultrasound-guided procedure required more time. Further studies are required to confirm these results in an in vivo sample and to analyze their clinical implications.

## Figures and Tables

**Table 1:** Baseline characteristics of the participants and overall data on interventions.

	Mean ± SD
Male/female (n)	4/1
Experience with invasive techniques (years)	10.00 ± 5.40
Experience with ultrasound (years)	8.80 ± 3.50
Total needle procedures (n)	100
Palpation-guided/ultrasound-guided (n)	50/50
With/without handpiece (n)	50/50
Distance to the target (mm)	1.24 ± 2.10
Ulnar nerve punctured (no/yes)	90/10
Distance of ulnar nerve punctured (mm)	0.22 ± 0.83
Success/failure (n)	81/19
Time required (s)	31.45 ± 21.71
Passes (total number)	1.97 ± 1.22
Needle length outside (mm)	19.35 ± 3.60

Abbreviations: n, number; mm, millimeters; s, seconds; SD, standard deviation.

**Table 2:** Measurements (mean, standard deviation) with palpation-guided and ultrasound-guided interventions.

	Palpation-Guided	Ultrasound-Guided	
	Mean ± SD	Mean ± SD	*p*
Distance to the target (mm)	2.01 ± 2.41	0.48 ± 1.37	<0.001
Ulnar nerve punctured (no/yes)	40/10	50/0	0.001
Distance of ulnar nerve punctured (mm)	0.44 ± 1.13	0 ± 0	0.007
Success/failure (n)	33/17	48/2	<0.001
Time required (s)	24.57 ± 17.84	38.33 ± 23.19	0.001
Passes (total number)	2.06 ± 1.11	1.88 ± 1.32	0.463
Needle length outside (mm)	19.82 ± 2.66	18.88 ± 4.32	0.193

Abbreviations: n, number; mm, millimeters; s, seconds; SD, standard deviation.

**Table 3:** Measurements (mean, standard deviation) with needle alone or with percutaneous electrolysis handpiece.

	With Handpiece	Needle	
	Mean ± SD	Mean ± SD	*p*
Distance to the target (mm)	1.56 ± 2.63	0.92 ± 1.34	0.127
Ulnar nerve punctured (no/yes)	43/7	47/3	0.318
Distance of ulnar nerve punctured (mm)	0.37 ± 1.11	0.07 ± 0.29	0.069
Success/Failure (n)	38/12	43/7	0.308
Time required (s)	29.41 ±16.01	33.48 ± 26.23	0.351
Passes (total number)	1.96 ± 1.14	1.98 ± 1.30	0.935
Needle length outside (mm)	18.24 ± 4.64	20.46 ± 1.46	0.002

Abbreviations: n, number; mm, millimeters; s, seconds; SD, standard deviation.

**Table 4:** Results of the self-perceived questionnaire.

	Palpation-Guided	Ultrasound-Guided	
Task question	**Mean ± SD**	**Mean ± SD**	***p***
Mental demand	1.20 ± 1.79	2.00 ± 2.45	0.655
Physical demand	0.40 ± 0.89	0.40 ± 0.89	1.000
Temporal demand	1.20 ± 2.68	1.20 ± 1.10	0.705
Performance	7.60 ± 1.82	8.80 ± 1.64	0.416
Effort	1.60 ± 3.58	0.80 ± 1.79	0.317
Frustration level	4.40 ± 2.97	2.00 ± 4.47	0.498
Learner’s assessment of their own ability, a	7.60 ± 2.30	8.20 ± 1.92	0.586
Preferred modality of guidance, b	0/5 (0%) PG vs. 5/5 (100%) UG		
Preferred modality of needle grip, b	0/5 (0%) with vs. 5/5 (100%) without handpiece		

Abbreviations: PG, palpation-guided; UG, ultrasound-guided; SD, standard deviation; a, from 1 (low) to 10 (high); b, number of participants.

## Data Availability

The data presented in this study are available on request from the corresponding author.
